# Performance of four cardiac output monitoring techniques vs. intermittent pulmonary artery thermodilution during a modified passive leg raise maneuver in isoflurane-anesthetized dogs

**DOI:** 10.3389/fvets.2023.1238549

**Published:** 2023-09-14

**Authors:** Vaidehi V. Paranjape, Natalia Henao-Guerrero, Giulio Menciotti, Siddharth Saksena

**Affiliations:** ^1^Department of Small Animal Clinical Sciences, Virginia-Maryland College of Veterinary Medicine, Virginia Polytechnic Institute and State University, Blacksburg, VA, United States; ^2^Department of Civil and Environmental Engineering, Virginia Polytechnic Institute and State University, Blacksburg, VA, United States

**Keywords:** arterial pressure waveform analysis, canine, electrical cardiometry, esophageal doppler, general anesthesia, fluid responsiveness, hypovolemia, transesophageal echocardiography

## Abstract

**Objective:**

This study investigated the performance among four cardiac output (CO) monitoring techniques in comparison with the reference method intermittent pulmonary artery thermodilution (iPATD) and their ability to diagnose fluid responsiveness (FR) during a modified passive leg raise (PLR_M_) maneuver in isoflurane-anesthetized dogs undergoing acute blood volume manipulations. The study also examined the simultaneous effect of performing the PLR_M_ on dynamic variables such as stroke distance variation (SDV), peak velocity variation (PVV), and stroke volume variation (SVV).

**Study design:**

Prospective, nonrandomized, crossover design.

**Study animals:**

Six healthy male Beagle dogs.

**Methods:**

The dogs were anesthetized with propofol and isoflurane and mechanically ventilated under neuromuscular blockade. After instrumentation, they underwent a series of sequential, nonrandomized steps: Step 1: baseline data collection; Step 2: removal of 33 mL kg^−1^ of circulating blood volume; Step 3: blood re-transfusion; and Step 4: infusion of 20 mL kg^−1^ colloid solution. Following a 10-min stabilization period after each step, CO measurements were recorded using esophageal Doppler (ED_CO_), transesophageal echocardiography (TEE_CO_), arterial pressure waveform analysis (APWA_CO_), and electrical cardiometry (EC_CO_). Additionally, SDV, PVV, and SVV were recorded. Intermittent pulmonary artery thermodilution (iPATD_CO_) measurements were also recorded before, during, and after the PLR_M_ maneuver. A successful FR diagnosis made using a specific test indicated that CO increased by more than 15% during the PLR_M_ maneuver. Statistical analysis was performed using one-way analysis of variance for repeated measures with *post hoc* Tukey test, linear regression, Lin’s concordance correlation coefficient (ρc), and Bland–Altman analysis. Statistical significance was set at *p* < 0.05.

**Results:**

All techniques detected a reduction in CO (*p* < 0.001) during hemorrhage and an increase in CO after blood re-transfusion and colloid infusion (*p* < 0.001) compared with baseline. During hemorrhage, CO increases with the PLR_M_ maneuver were as follows: 33% for iPATD (*p* < 0.001), 19% for EC (*p* = 0.03), 7% for APWA (*p* = 0.97), 39% for TEE (*p* < 0.001), and 17% for ED (*p* = 0.02). Concurrently, decreases in SVV, SDV, and PVV values (*p* < 0.001) were also observed. The percentage error for TEE, ED, and EC was less than 30% but exceeded 55% for APWA. While TEE_CO_ and EC_CO_ slightly underestimated iPATD_CO_ values, ED_CO_ and APWA_CO_ significantly overestimated iPATD_CO_ values. TEE and EC exhibited good and acceptable agreement with iPATD. However, CO measurements using all four techniques and iPATD did not differ before, during, and after PLR_M_ at baseline, blood re-transfusion, and colloid infusion.

**Conclusion and clinical relevance:**

iPATD, EC, TEE, and ED effectively assessed FR in hypovolemic dogs during the PLR_M_ maneuver, while the performance of APWA was unacceptable and not recommended. SVV, SDV, and PVV could be used to monitor CO changes during PLR_M_ and acute blood volume manipulations, suggesting their potential clinical utility.

## 1. Introduction

Circulatory shock can pose as a life-threatening event, occurring because of inadequate blood flow and oxygen delivery to tissues. Amidst such hemodynamic instability, the cornerstone of treatment lies in fluid therapy, which ameliorates venous return, cardiac preload, cardiac output (CO), and tissue oxygen utilization (1). Over the past two decades, the concept of “fluid responsiveness” (FR), rooted in the Frank-Starling cardiac curve, has garnered significant attention, targeting the challenges of precisely dosing fluid therapy. Following a fluid bolus, an increase of more than 10–15% in CO or stroke volume signifies FR, as there are heightened cardiac preload and ventricular filling pressures. By contrast, minimal changes occur with fluid nonresponsiveness (2–5). FR evaluation uses static or dynamic indices. Static indices such as central venous pressure and pulmonary artery occlusion pressure, although established as cardiac filling pressures, lack a strong correlation with FR (6, 7). Dynamic indices such as stroke volume variation (SVV) and pulse pressure variation depend on heart-lung interactions induced by mechanical ventilation and measure the subsequent effect of positive-pressure ventilation on fluctuations in stroke volume across a respiratory cycle. While these indices hold clinical relevance in both human medicine (2–4) and veterinary medicine (8, 9), their accuracy diminishes during spontaneous breathing, low tidal volume ventilation, cardiac rhythm disturbances, right-sided heart failure, and open chest conditions (2, 10, 11). In such scenarios, researchers have introduced the “passive leg raising” test that can trigger a preload challenge similar to a traditional fluid bolus or heart-lung interactions, effectively predicting FR based on multiple trials and meta-analyses (12–15).

Historically, passive leg raising was employed as an emergency measure during acute circulatory crises or hemorrhagic events in human patients. This simple bedside test shifts a patient from a semi-recumbent to a horizontal trunk position, raising the lower limbs at 30–45° (2, 16, 17). This gravitational shift causes the movement of approximately 150–300 mL of blood volume from the lower body to cardiac chambers, mimicking a fluid bolus’ effects (18, 19). This transient and reversible hemodynamic change eliminates the risk of volume overload. In an ideal scenario, real-time cardiovascular assessment is crucial to capture brief circulatory adjustments during passive leg raising (2, 16, 17). Transthoracic or transesophageal echocardiography has been routinely used in diverse human populations to precisely monitor the real-time cardiovascular response to this test (2, 12–14, 16). A replication of the 45° passive leg raising test during cardiopulmonary resuscitation in an experimental porcine model of prolonged ventricular fibrillation yielded comparable outcomes to those of standard positioning in terms of spontaneous circulation return and 24-h survival rates. Animals undergoing passive leg raising exhibited notably better neurological alertness scores (20).

Until recently, only limited literature has been available on this maneuver in veterinary species. Recognizing inter-species variations in pelvic limb conformation, size, and blood volume distribution, a modified passive leg raise (PLR_M_) maneuver was developed and demonstrated in anesthetized pigs (21) and dogs (22). During acute hemorrhage, the inclination of the pelvic limbs and caudal abdomen at 15° in pigs and 30° in dogs caused a significant CO increase of more than 25%, measured through intermittent pulmonary artery thermodilution (iPATD_CO_). While iPATD_CO_ is often regarded as the “gold standard” for veterinary research (23–25), its application in clinical settings is hindered by complications, expertise requirements, extensive training, and costly equipment setup (26). Real-time minimally invasive (e.g., esophageal Doppler [ED_CO_], transesophageal echocardiography [TEE_CO_], arterial pressure waveform analysis [APWA_CO_]) or noninvasive (e.g., electrical cardiometry [EC_CO_]) methods that provide continuous, reproducible CO measurements remain unvalidated for assessing PLR_M_ in animals.

The objectives of the present study were as follows: (1) gaging the agreement between invasive iPATD_CO_ and ED_CO_, TEE_CO_, APWA_CO_, and EC_CO_ for monitoring CO changes in response to PLR_M_ in anesthetized dogs subjected to hemodynamic effects from induced blood volume changes; (2) evaluating the ability of ED_CO_, TEE_CO_, APWA_CO_, and EC_CO_ to identify more than 15% iPATD_CO_ increase, indicative of FR; and (3) investigating the correlation between EC and ED-derived dynamic variables like SVV, stroke distance variation (SDV), and peak velocity variation (PVV) with iPATD_CO_ measurements during PLR_M_ analysis. The following hypotheses were proposed: (1) strong and acceptable agreement will be observed between ED_CO_, TEE_CO_, APWA_CO_, EC_CO_, and iPATD_CO_; (2) all test methods will detect more than 15% increase in CO signifying FR; and (3) SVV, SDV, and PVV will demonstrate correlation with iPATD_CO_ readings during PLR_M_.

## 2. Materials and methods

### 2.1. Ethics statement

This prospective, nonrandomized, crossover experimental study involving dogs was reviewed and granted approval by the Institutional Animal Care and Use Committee of Virginia Tech University (protocol number 20-235).

### 2.2. Animals in the study

Six adult, sexually intact male, healthy Beagles aged 13–16 months and weighing 10.5 ± 0.3 kg (mean ± standard deviation), bred specifically for research, were included in this investigation. A comprehensive physical examination, complete blood count, and serum chemistry panel were conducted on all dogs. The dogs were deemed healthy as no abnormalities were observed in the blood test results or during the cardiopulmonary examination. Following an extensive review of pertinent literature on human studies detecting a positive CO response to the passive leg raising test (2, 12–15), published data on the PLR_M_ maneuver in pigs (21), and an unpublished pilot study involving dogs, an *a priori* power analysis affirmed that a sample size of at least six dogs would be necessary to detect a significant 15% difference in CO, assuming a statistical power of 0.8 and an alpha level of 0.05 (G*Power 3.1, Heinrich-Heine-Universität Düsseldorf, Germany). An effect size greater than 0.8, derived from these animal studies, indicated a substantial difference effect. The dogs were individually housed in kennels under controlled temperature conditions and were acclimatized to the laboratory environment for 2 weeks. Food was withheld for 12 h before the experimental procedures, while access to water was maintained. A physical examination was conducted for all dogs on the day of general anesthesia.

### 2.3. Anesthetic induction and standard anesthetic monitoring

The determination of the order in which each dog underwent the experiment on a given day was randomized using a tool available at: https://www.randomizer.org/. On the designated study day, intravenous (IV) catheterization of the right cephalic vein was performed. This was followed by preoxygenation using a facemask to administer oxygen at a flow rate of 4Lmin^−1^. General anesthesia was induced facemask to administer IV propofol (Propoflo 10 mg mL^−1^, Zoetis Inc., MI, United States), titrated until orotracheal intubation could be accomplished with an appropriately sized, cuffed endotracheal tube. After securing the airway, the dog was connected to an anesthesia workstation integrated with a ventilator (Datex-Ohmeda Aestiva 5/7900, GE Healthcare, WI, United States) through a circle anesthetic breathing circuit. Anesthetic maintenance was achieved using isoflurane (Fluriso, VetOne, ID, United States) in oxygen (1–2 L min^−1^), targeting an end-tidal concentration of isoflurane between 1.5 and 1.7%. The dogs were placed in a right lateral recumbent position. End-tidal isoflurane concentration and end-tidal carbon dioxide concentration were continuously monitored using a calibrated side-stream infrared gas analyzer linked to a multiparameter monitor (Datex-Ohmeda S/5 Compact anesthesia monitor; GE Healthcare). Using the same monitor, Standard lead II electrocardiogram, heart rate (HR), esophageal temperature, and peripheral hemoglobin oxygen saturation were also recorded at 5-min intervals throughout the experiment, as part of routine anesthetic monitoring and record keeping. Body temperature was maintained between 36.7°C and 38.1°C during anesthesia using a forced-air warming blanket and a circulating water-heating system.

Arterial catheterization of the dorsal pedal artery was performed to measure invasive systolic, diastolic, and mean arterial blood pressures. The catheter was connected to a disposable pressure transducer through non-compliant short tubing (15.24 cm) filled with heparinized saline (2 IU mL^−1^) and connected to a three-way Luer lock stopcock. The transducer was positioned at a height approximating the right atrium location. Neuromuscular paralysis was initiated to prevent patient-ventilator dyssynchrony, with an initial IV dose of 0.4 mg kg^−1^ rocuronium (rocuronium bromide 10 mg mL^−1^, Pfizer, NY, United States), followed by a continuous rate infusion of 0.4 mg kg^−1^ h^−1^. Supramaximal stimulation of the common peroneal nerve was generated using a peripheral nerve stimulator (Stimpod 450X, Xavant Technology, Pretoria, SA) to assess the effectiveness of the blockade. Mechanical ventilator settings were adjusted to a volume-controlled mode set at 12 mL kg^−1^, with an inspiratory-to-expiratory ratio ranging from 1:2 to 1:3, and the respiratory rate was adjusted to maintain end-tidal carbon dioxide concentration between 30 and 40 mmHg. Throughout the experiment, no maintenance crystalloid fluids were administered to prevent potential blood volume changes that could skew hemodynamic data.

### 2.4. Instrumentation for EC_CO_ measurements

The technique employed by the ICON monitor (Osypka Medical Inc., CA, United States) for continuous hemodynamic data monitoring, including EC_CO_ and SVV readings, is referred to as electrical cardiometry. After preparing the skin areas by clipping and cleaning, four Cardiotronic (Osypka Medical Inc.) electrocardiographic electrodes with adhesive patches were positioned. Two electrodes were placed on the left aspect of the neck (at the level of the common carotid artery), and the other two electrodes were attached to the left thoracic area (at the level of the T8-T13 vertebrae), as previously described in published studies (27–29). These electrodes were then connected to the ICON EC monitor through a cable. The ICON EC monitor was synchronized with a laptop using the iControl^™^ software application (Osypka Medical Inc.) and an external communication cable, facilitating efficient data management. The ICON monitor employed the Electrical Velocimetry^™^ (Osypka Medical Inc.) physiological model to assess changes in thoracic electrical bioimpedance during cardiac systole, thereby capturing volumetric changes in the aorta. Detailed information on the physiology and algorithmic calculations used for EC_CO_ derivation can be found in other studies (28, 29). Additionally, the computation of SVV was automated with ICON internal software using a specific formula.


$$SVV\;\left(\%\right)=\frac{{SV}_{\text{max}}-{SV}_{\text{min}}}{\left({SV}_{\text{max}}+{SV}_{\text{min}}\right)/2}\;\times \;100$$

where SV_max_ and SV_min_ are the maximum and minimum stroke volume (mL), respectively, over one respiratory cycle.

At each data point, the EC_CO_ (L min^−1^) and SVV (%) data were recorded as an averaged value over a 1-min interval, as configured in the internal database settings.

### 2.5. Instrumentation for iPATD_CO_ measurements

Subsequently, the dogs were positioned in dorsal recumbency and maintained in this posture throughout the course of the experiment. Areas of approximately 4 × 4 cm over the left and right jugular veins were clipped and prepared with aseptic techniques. A 5 Fr 13-cm double-lumen central venous catheter (MILA International Inc., KY, United States) was introduced into the left jugular vein to draw a consistent blood volume during acute hemorrhagic shock and to facilitate blood transfusion and colloid solution administration during correction of hypovolemia. Concurrently, a 6 Fr 8.5 cm hemostasis introducer (Fast-Cath, Abbott Cardiovascular, MN, United States) was positioned in the right jugular vein, through which a 5 Fr 75 cm pulmonary artery Swan Ganz catheter (132FS, Edwards Lifesciences Corp., CA, United States) was inserted. Both the proximal and distal ports of the pulmonary artery catheter were connected to additional disposable pressure transducers. These transducers were calibrated and positioned in a manner analogous to the arterial pressure transducer. The pulmonary artery catheter was cautiously advanced through the right atrium and right ventricle until its distal port was situated within the pulmonary artery. The positioning accuracy was affirmed by monitoring characteristic pressure waveforms and values, using the CO monitor (Carescape B850, GE Healthcare, IL, United States). Measurement of the pulmonary artery wedge aided the assessment of left ventricular filling and left atrial pressure, pertaining to a concurrent independent research study. For the determination of iPATD_CO_, a 3-mL chilled (2–5°C) bolus of 0.9% sodium chloride solution was injected at the end of the expiration phase for <3 s through the proximal injectate port. The appropriate computation constant was selected on the CO monitor screen based on the catheter model, injectate volume, and temperature, as advised by the manufacturers. The catheter thermistor measured core body temperature as well as the difference in blood temperature downstream, which the CO monitor converted into a dilution curve through a modified Stewart-Hamilton equation. At each data timepoint, a CO reading represented the mean of three consecutive measurements within a 10% variation range. The injections were manually executed consistently by the same individual, with a minimum interval of 90 s between each injection.

### 2.6. Instrumentation for arterial pressure waveform analysis-based CO measurements

The LiDCOplus monitor (LiDCO Ltd., Cambridge, UK) employed the PulseCO algorithm based on pulse power analysis to continuously determine real-time CO values. This algorithm relied on arterial pressure waveforms and lithium dilution for intermittent calibration. To calibrate, a 0.006 mmol kg^−1^ IV bolus of lithium chloride (LiDCO Ltd.) was injected through the central venous catheter in the left jugular vein. The concentration of lithium was measured by a lithium ion-sensitive electrode sensor connected to a three-way stopcock on the indwelling arterial catheter (30, 31). Blood samples were collected using a peristaltic pump at a flow rate of 4 mL min^−1^ across the sensor. The packed cell volume and serum sodium concentration, which were correction factors required by the LiDCO monitor, were analyzed using a benchtop blood gas machine just before CO measurement. The resultant lithium concentration vs. time was employed to calculate plasma flow using the Stewart-Hamilton equation. The calibration CO value (L min^−1^) was determined using the following formula (32, 33):


$$\text{LiDCO}\;\left(L/\min\right)=\frac{\text{Lithium}\;\text{chloride}\;\text{dose}\;\left(\text{mmol}\right)\;\times \;60}{\begin{array}{l} \text{area}\;\text{of}\;\text{curve}\;\text{corrected}\;for\;\text{sodium}\;\\ \;\text{concentration}\;\left(\text{mmol}\;L-1\right)\;\times \;\\ \;\left(1-\text{packed}\;\text{cell}\;\text{volume}\right) \end{array}}$$

The PulseCO algorithm integrated a time-based autocorrelation and transformed the arterial waveform into a volume-time waveform, accounting for compliance and aortic volume. The root mean square method, independent of waveform morphology, estimated the effective value (approximately 0.7 times the original amplitude) of this volume waveform, calculating the “nominal stroke volume.” This was then scaled to an “actual stroke volume” using a patient-specific calibration derived from the lithium dilution CO measurement, considering the individual’s age, height, and weight (33–35). The APWA_CO_ (arterial pressure waveform analysis-based CO) was automatically calculated as the product of stroke volume and heart rate (HR), continuously monitored and recorded by the LiDCOplus monitor. At each data point, the recorded value for PulseCO was an average over a 1-min interval.

### 2.7. Instrumentation for TEE_CO_ measurements

A multiplane transesophageal transducer (Canon i6SVX2–1.8-6mHz, Canon Medical Systems, CA, United States) was inserted into the esophagus, reaching a mid-esophageal position. The image plane was digitally rotated forward until a mid-esophageal long-axis view was obtained (36, 37). The image was optimized for clear visualization of the left ventricular outflow tract (LVOT), the sinus of Valsalva, and the proximal portion of the ascending aorta. The “zoom” function of the ultrasound machine (Aplio i900, Canon Medical Systems, CA, United States) was employed to enhance the visibility of these structures. Electrocardiogram-triggered cine-loop, encompassing three consecutive cardiac cycles, was stored for subsequent offline analysis. Subsequently, the probe was advanced to a deep trans-gastric position and its tip was anteflexed to obtain a short-axis view of the left ventricle. The image plane was further rotated forward to visualize the LVOT in a position as parallel as possible to a Doppler gate. This image plane remained fixed for the rest of the procedure. At predetermined time points, a pulsed wave-Doppler gate was positioned in the LVOT, just below the aortic valve, in accordance with the American Society of Echocardiography Guidelines (38). Care was exercised to position the gate at the same location where the LVOT measurements were taken. Spectral Doppler traces spanning at least three cardiac cycles were stored for subsequent analysis. Continuous-wave Doppler was employed in a similar manner when the Nyquist limit was reached to acquire LVOT Doppler spectrograms. The acquired images and cine-loops were imported into a workstation equipped with specialized cardiac analysis software (Tomtec Arena, Tomtec Imaging Systems, Germany). All measurements were conducted over three consecutive cardiac cycles, and the average values were used for statistical analysis. LVOT diameter was measured from zoomed images captured from the mid-esophageal long-axis view, selecting a mid-systolic frame just below the aortic valve. The LVOT cross-sectional area (LVOTArea) was then calculated as **π** × (LVOT diameter/2)^2^. To minimize sources of error, given that LVOTArea was not anticipated to vary significantly over the study duration, the LVOTArea was measured only once for each dog (38). For each acquired Doppler spectrogram, the velocity time integral of three consecutive beats was measured using the embedded function of the software program. Instantaneous heart rate (HR) was also measured by the embedded function of the software, calculating the time between two consecutive envelope peaks. At each time point, TEE_CO_ was calculated as follows: velocity time integral × LVOTArea × HR.

### 2.8. Instrumentation for ED_CO_ measurements

The esophageal Doppler veterinary monitoring system (CardioQ-EDMV+, Deltex Medical, UK), equipped with a 4.02-MHz continuous Doppler ultrasound emitting probe (K9P, Deltex Medical), was utilized to estimate stroke distance (cm), SDV (%), and PVV (%). The probe, measuring 120 cm in length, featured 6 depth markers to aid in guiding the insertion depth and ensuring proper probe placement. Following lubrication with aqueous gel, the probe was introduced into the oral cavity alongside the existing TEE probe and gently advanced into the esophagus. Further insertion continued until the probe tip reached a position approximately aligned with the fifth to sixth thoracic vertebra, coinciding with peak aortic blood flow. Once a characteristic triangular waveform, accompanied by a distinct maximal pitch Doppler “whip crack” sound, was visualized, the probe was stabilized in that position to confirm peak blood flow from the descending thoracic aorta. The working principle of this method, Doppler waveform analysis, and the derived variables have been recently documented in canine studies (25). The CardioQ-EDMV+ monitor did not automatically display quantified ED_CO_ values. Therefore, the LVOTArea measured using TEE was used to calculate ED_CO_ (L min^−1^) with the following formula, referencing a study conducted in canines (39):


$$\begin{array}{l} {ED}_{CO}\;\left(L/\min\right)=\text{LVOTArea}\;\left({\text{cm}}^{2}\right)\times \text{Stroke}\;\text{distance}\;\left(\text{cm}\right)\\ \;\times HR\;\left(\text{beats}\;\min^{-1}\right) \end{array}$$

During each mechanical breath delivered by the ventilator, SDV and PVV were automatically computed by an internal software program, and the results were continuously displayed on the CardioQ-EDMV+ monitor screen (25). Before data collection at each time point, a minimum of 3 min was allowed to ensure a consistent, high-quality signal and Doppler waveform. The average values for stroke distance, SDV, and PVV over a 1-min cycle were recorded.


$$SDV\;\left(\%\right)=\frac{\text{Stroke}\;{\text{Distance}}_{\text{max}}-\text{Stroke}\;{\text{Distance}}_{\text{min}}}{\left(\text{Stroke}\;{\text{Distance}}_{\text{max}}+\text{Stroke}\;{\text{Distance}}_{\text{min}}\right)/2}\;\times \;100$$

$$PVV\;\left(\%\right)=\frac{{PV}_{\text{max}}-{PV}_{\text{min}}}{\left({PV}_{\text{max}}+{PV}_{\text{min}}\right)/2}\;X\;100$$

### 2.9. Study timeline and performing PLR_M_ maneuver

**Figure 1:**
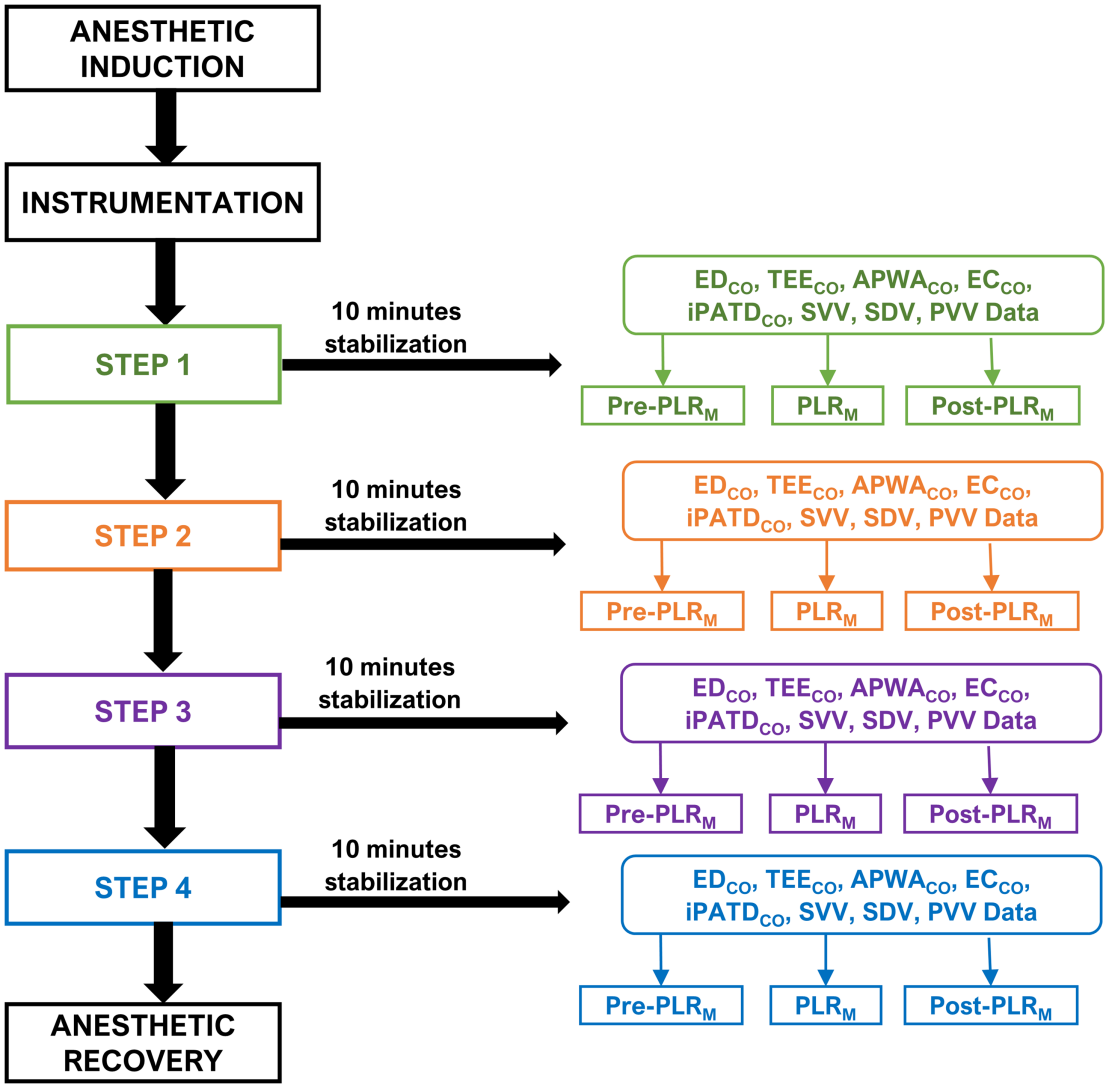
Timeline of the experimental study design and cardiac output data: The timeline of this experimental study design and cardiac output data was obtained through various techniques such as minimally invasive esophageal Doppler (ED_CO_), transesophageal echocardiography (TEE_CO_), arterial pressure waveform analysis (APWA_CO_), noninvasive electrical cardiometry (EC_CO_), and the gold standard intermittent pulmonary artery thermodilution (iPATD_CO_). The evaluation was conducted during a modified passive leg raise (PLR_M_) maneuver in six healthy anesthetized Beagle dogs. After anesthesia induction and instrumentation, each dog underwent a sequential nonrandomized four-step sequence: Step 1: baseline data collection; Step 2: induction of hypovolemia by withdrawing 33 mL kg^−1^ of circulating blood volume over a 15-min period; Step 3: autologous blood transfusion over 15 min; and Step 4: administering a 20 mL kg^−1^ intravenous bolus of a colloid solution over 15 min. A 10-min stabilization period was implemented after each step and before data collection. In each step, hemodynamic data were collected three times: before the PLR_M_ maneuver (Pre-PLR_M_); after 5 min of being in the PLR_M_ maneuver, wherein the elevation of the pelvic limbs and caudal abdomen was assessed at 30° (PLR_M_); and after 5 min of returning to the original position (Post-PLR_M_). The final data were obtained, the jugular and arterial catheters were removed, and anesthetic recovery was initiated.

After completing the instrumentation process and confirming the proper functioning of all CO equipment, each dog underwent a sequential nonrandomized study design to induce acute alterations in blood volume. The experiment followed a four-step sequence (Figure 1): Step 1 involved obtaining baseline data corresponding to euvolemia; Step 2 initiated acute hemorrhage by withdrawing 33 mL kg^−1^ of circulating blood volume from the left jugular catheter over a 15-min period (induction of hypovolemia). The removed blood was stored in blood collection bags coated with citrate phosphate dextrose adenine as an anticoagulant. Step 3 encompassed autologous blood transfusion through the left jugular catheter over 15 min using an infusion pump (to restore lost blood volume); Step 4 comprised the administration of a 20 mL kg^−1^ IV bolus of 6% hydroxyethyl starch (VetStarch 130/0.4 in 0.9% sodium chloride, Zoetis Inc.) as a colloid solution into the left jugular vein over a 15-min period (induction of hypervolemia). After each step, a 10-min period was provided for hemodynamic stabilization. The purpose of subjecting the dogs to various blood volume phases was to induce significant CO changes during each step, creating a physiological context to demonstrate and simultaneously assess the presence or absence of FR using the various CO techniques.

**Figure 2:**
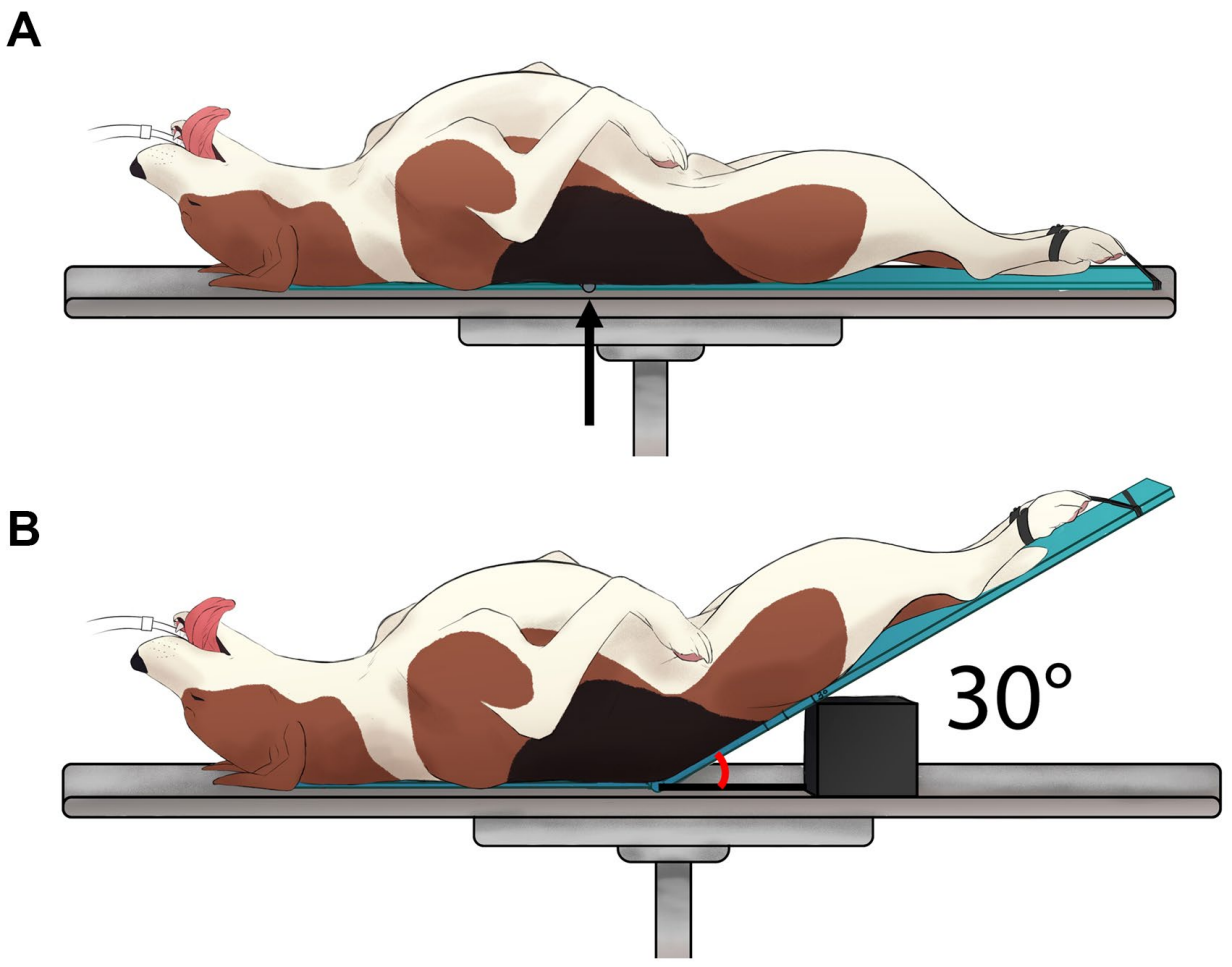
Graphical illustration of the modified passive leg raise (PLR_M_) maneuver: A visual depiction of the PLR_M_ maneuver that was performed in six healthy, anesthetized dogs according to the detailed description published recently in a canine model (22). The PLR_M_ maneuver involved two states: **(A)** Before performing the PLR_M_ maneuver, and **(B)** the PLR_M_ maneuver itself, achieved by raising the caudal abdomen and pelvic limbs at 30° relative to the horizontal surface of the table [Created by V. Paranjape and J. Mauragis]. This image is reproduced with permission from Source: Paranjape et al. (22).

The Passive Leg Raise Maneuver (PLR_M_), as depicted in Figure 2, was executed according to the recently characterized detailed description in dogs (22). Hemodynamic data were collected under each step three times: before performing the PLR_M_ maneuver (Pre-PLR_M_; see Figure 2A), after 5 min of the PLR_M_ maneuver involving elevating the pelvic limbs and caudal abdomen at a 30° angle (PLR_M_; Figure 2B), and after returning the original position for 5 min (Post-PLR_M_; Figure 2A).

### 2.10. Summary for data collection

To ensure unbiased data collection, each researcher was assigned to collect data from a specific CO monitoring technique and remained unaware of the data recorded by others. One researcher was responsible for altering body positions during the PLR_M_ maneuver. While the order for collecting CO data from the four test methods in the study (EC, APWA, TEE, and ED) was randomized (using https://www.randomizer.org/), iPATD_CO_ measurements were consistently performed after acquiring CO data from the other CO techniques. This sequencing was designed to prevent the fluid volume from multiple 0.9% saline injections for iPATD_CO_ potentially affecting data from the test methods. Moreover, preserving the required sodium concentrations for LiDCOplus CO calibration in determining APWA_CO_ values was essential, which could be influenced by the administration of 0.9% saline injections. Calibration of CO by the LiDCOplus monitor for estimating APWA_CO_ values was conducted before Step 1, Step 3, and Step 4. The data recorded under each step for studying the effects of the PLR_M_ maneuver (Pre-PLR_M_, during PLR_M_, and Post-PLR_M_) are summarized as follows:

- ICON electrical cardiometry monitor measured EC_CO_ and SVV values.
- LiDCOplus monitor with the PulseCO algorithm for pulse power analysis- measured APWA_CO_ values.
- Transesophageal echocardiography probe measured velocity time integral and LVOTArea to calculate TEE_CO_ measurements.
- CardioQ-EDMV+ esophageal Doppler monitor measured stroke distance, SDV, and PVV values.
- Carescape B850 intermittent pulmonary artery thermodilution monitor measured iPATD_CO_ values.

### 2.11. Anesthetic recovery

Subsequent to the final data collection, rocuronium administration was ceased. A train-of-four ratio ≥0.9, consistent spontaneous ventilation, and generation of more than 3 cmH_2_O negative pressure during spontaneous inspiration were taken as indicators of recovery from rocuronium-induced neuromuscular block. The pulmonary artery, jugular, and arterial catheters were removed with careful application of external pressure on the catheter sites to prevent bleeding and hematoma formation. Anesthetic recovery was marked by discontinuing the isoflurane vaporizer. Following extubation, all dogs received 0.3 mg kg^−1^ IV methadone (methadone hydrochloride injection 10 mg mL^−1^, Akorn, IL, United States), and they were transferred to individual kennels. Cardiopulmonary parameters were monitored, and catheter sites were periodically observed over the subsequent 96 h. Pain scores were assessed using the Glasgow composite pain scale short form, along with physical examination and noninvasive blood pressure measurements. Additional administration of 0.25 mg kg^−1^ IV methadone was considered if needed based on pain scores.

### 2.12. Statistical analysis

The normality of variables across the sequential steps (baseline, hemorrhage, autologous blood transfusion, and hydroxyethyl starch infusion) for each CO measurement method (iPATD, EC, APWA, TEE, and ED) was assessed using the Shapiro–Wilk and D’Agostino-Pearson tests. All variables were determined to be normally distributed and are presented as mean ± standard deviation. A one-way analysis of variance for repeated measures was employed to compare differences between the sequential steps during acute blood volume manipulation for all variables, with the Greenhouse and Geisser correction applied when sphericity assumptions were not met. *Post hoc* Tukey adjustment was applied for multiple pairwise comparisons. Student *t*-tests were also conducted for pairwise comparisons, with significance set at *p* < 0.05 for all analyses. According to the definition of FR (2–5), the test methods were deemed successful in diagnosing FR in response to the PLR_M_ only if there was a more than 15% increase in CO values during the PLR_M_ maneuver compared to Pre-PLR_M_ CO values.

To calculate bias, the difference between iPATD_CO_ and CO values for each of the other methods (EC_CO_, APWA_CO_, TEE_CO_, and ED_CO_) was expressed as a percentage of the average CO values between iPATD_CO_ and the specific method. This relative bias was represented as a percentage of average CO values, utilizing a formula previously reported (24). Relative bias was considered positive when CO values were underestimated by other methods in comparison to iPATD_CO_, and negative when CO values were overestimated. Limits of agreement were calculated as relative bias ±1.96 × standard deviation to encompass 95% confidence intervals. The absolute value of relative bias for each observation was compared with a 30% threshold to calculate the count of observations within the acceptable performance range. The percentage error was expressed as 1.96 × standard deviation of bias/mean CO × 100, calculated for all test methods, and compared with the acceptable range (<30%) proposed in the literature (40).

The relationships between the reference method iPATD_CO_ and test methods (iPATD, EC, APWA, TEE, and ED) were analyzed using linear regression, and concordance was assessed using Lin’s concordance correlation coefficient (ρc), where perfect concordance equated to a value of 1 (41). Agreement between iPATD_CO_ and individual test methods was evaluated through the Bland–Altman (BA) method (42), and a polar plot was employed to visualize the agreement (43, 44). In cases where bias between CO values of test methods and reference (iPATD_CO_) depended on the magnitude of the original measurement, a BA analysis for nonuniform differences was performed, and the bias was regressed linearly against the average bias. The distance from the center indicated the absolute values of the mean change in ([∆CO_PATD_+ ∆CO_EC_]/2), while the angle with the horizontal (0° radial) indicated disagreement. A good trend was evident when data were situated within 10% of mean CO values. Commercial statistical software (SAS version 9.4; SAS Institute Inc.) was utilized for all analyses, with BA and polar plots generated using available software (Excel, Microsoft Corp.; Polar Plot 2 analysis add-in; accessed 11 December, 2022)^1^.

## 3. Results

The anesthetic induction, maintenance, and recovery phases proceeded without adverse events for all dogs. In each case, iPATD instrumentation and placement of the thermodilution catheter were successfully executed without any reported complications. No missing data were noted for iPATD, EC, APWA, TEE, and ED. The experimental procedures were completed successfully across all dogs. The dogs maintained normothermia throughout the anesthetic period (37.2 ± 0.6°C). No discernible differences were noted in total anesthesia time (*p* = 0.51), time spent during hemorrhage (*p* = 0.46), time spent during blood transfusion (*p* = 0.89), and colloid administration (*p* = 0.66) among the dogs.

Compared with baseline Pre-PLR_M_ CO values, significant reductions (*p* < 0.001) in hemorrhage Pre-PLR_M_ CO values were detected by iPATD, EC, APWA, TEE, and ED. Following blood transfusion and colloid infusion, Pre-PLR_M_ CO values were notably higher (*p* < 0.001) than baseline measurements, consistent across all test methods, including iPATD. Throughout baseline, blood transfusion, and colloid infusion, iPATD_CO_ measurements displayed no disparity between the Pre-PLR_M_, PLR_M_, and Post-PLR_M_ readings. This trend was mirrored in EC_CO_, APWA_CO_, TEE_CO_, and ED_CO_ measurements. During blood volume depletion, iPATD_CO_ showed an approximately 33% increase during the PLR_M_ maneuver as compared to Pre-PLR_M_ CO measurements. When evaluating the test methods, EC, APWA, TEE, and ED identified an approximate increase in PLR_M_ CO values of 19% (*p* = 0.03), 7% (*p* = 0.97), 39% (*p* < 0.001), and 17% (*p* = 0.02), respectively. Thus, alongside iPATD, only EC, TEE, and ED could successfully diagnose FR in response to the maneuver in hypovolemic dogs. Derived dynamic variables from EC (SVV) and ED (SDV and PVV) accurately tracked changes in CO values resulting from acute blood volume manipulation (Table 1). Commencement of acute hemorrhage induced significant increases in SVV, SDV, and PVV values (*p* < 0.001) compared with baseline. Following volume replacement with blood and colloids, these variables returned to baseline levels. However, no differences were observed between Pre-PLR_M_, PLR_M_, and Post-PLR_M_ readings for these variables during Steps 1, 3, and 4. By contrast, during hypovolemia, the maneuver led to a significant decrease in SVV, SDV, and PVV (*p* < 0.001) compared with Pre-PLR_M_ values. Returning the abdomen and legs to the horizontal position significantly increased these values, moving them closer to Pre-PLR_M_ readings (*p* < 0.001).

**Table 1:** Mean ± standard deviation of hemodynamic variables stroke volume variation (SVV), stroke distance variation (SDV), and peak velocity variation (PVV) derived from electrical cardiometry (EC) and esophageal Doppler (ED) monitors recorded in six healthy, mechanically ventilated, isoflurane-anesthetized dogs undergoing sequential manipulation of blood volume: Step 1: baseline; Step 2: acute hemorrhage by withdrawing circulating blood volume (33 mL kg^−1^); Step 3: autologous blood transfusion; and Step 4: 20 mL kg^−1^ intravenous bolus of 6% hydroxyethyl starch (colloid). Data were collected immediately before performing the modified passive leg raise (PLR_M_) maneuver (Pre-PLR_M_), after 5 min of PLR_M_ (PLR_M_), and 5 min after the abdomen and limbs were returned to the horizontal position (Post-PLR_M_).

Variable	Step	Time of data collection		
		Pre-PLR_M_	PLR_M_	Post-PLR_M_
SVV (%) derived from EC	1	9 ± 3	10 ± 2	10 ± 2
	2	22 ± 4^*^	15 ± 3^*, a^	25 ± 2^*, b^
	3	8 ± 2^†^	9 ± 1^†^	10 ± 1^†^
	4	6 ± 1^†^	5 ± 1^†^	5 ± 1^†^
SDV (%) derived from ED	1	10.4 ± 0.5	11.2 ± 0.3	11.5 ± 0.2
	2	19.2 ± 0.6^*^	12.7 ± 0.7^*, a^	21.4 ± 0.5^*, b^
	3	8.1 ± 0.8^†^	7.7 ± 0.6^†^	7.9 ± 0.7^†^
	4	5.5 ± 0.4^*, †^	5.1 ± 0.2^*, †^	5.4 ± 0.5^*, †^
PVV (%) derived from ED	1	6.2 ± 0.2	6.4 ± 0.5	6.5 ± 0.7
	2	16.8 ± 0.7^*^	9.8 ± 0.4^*, a^	14.1 ± 0.5^*, b^
	3	7.3 ± 0.8^†^	7.1 ± 0.5^†^	7.5 ± 0.7^†^
	4	4.4 ± 0.5^†^	4.1 ± 0.6^†^	3.8 ± 0.6^†^

^*^Significant difference from step 1 at the same time point (*p* < 0.05). ^†^Significant difference from step 2 at the same time point (*p* < 0.05).

^a^Significant difference from Pre-PLR_M_ within the same step (*p* < 0.05). ^b^Significant difference from PLR_M_ within the same step (*p* < 0.05).

SVV, Stroke Volume Variation; SDV, Stroke Distance Variation; PVV, Peak Velocity Variation.

**Figure 3:**
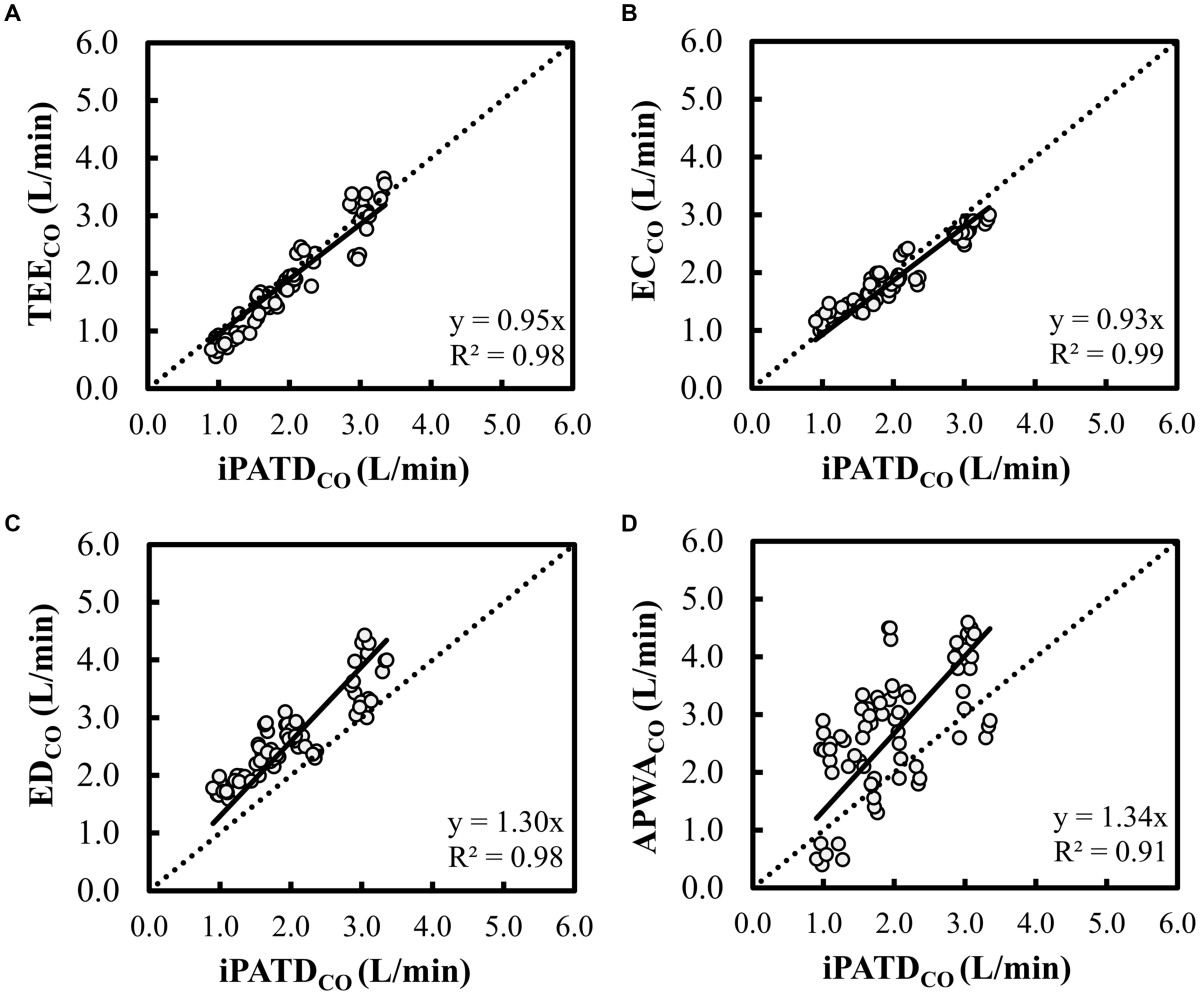
Scatterplot of cardiac output (CO) measurements using **(A)** transesophageal echocardiography (TEE_CO_), **(B)** electrical cardiometry (EC_CO_), **(C)** esophageal Doppler (ED_CO_), and **(D)** arterial pressure waveform analysis (APWA_CO_) against the reference method intermittent pulmonary artery thermodilution (iPATD_CO_) in six healthy, anesthetized dogs across three timepoints (before, during, and after the modified passive leg raise maneuver) and during sequential acute manipulations to the blood volume, thus yielding 72 paired observations (circles) per test method. The dotted line equates to the best-fit correlation. When compared with iPATD_CO_ measurements, TEE_CO_ and EC_CO_ displayed consistent slight underprediction (slope < 1 about Y = X on regression line), while ED_CO_ and APWA_CO_ showed constant significant overprediction of iPATD_CO_ measurements (slope > 1 about Y = X on regression line).

For each dog, pairs of CO measurements were obtained using iPATD and EC, APWA, TEE, and ED techniques at Pre-PLR_M_, PLR_M_, and Post-PLR_M_ timepoints, during baseline, hemorrhage, autologous blood transfusion, and colloid infusion. Thus, 12 pairs of CO comparisons between iPATD and the four test methods were collected for each dog, amounting to 72 pairs per test method across all six dogs. Percentage error, relative bias, LOA, and Lin’s concordance correlation coefficient for all test methods across all time points are presented in Table 2. TEE, ED, and EC exhibited percentage errors within the acceptable range (<30%) established as a standard (40), while APWA showed >55% error in performance across time points and during sequential manipulation of blood volume. Lin’s concordance correlation, indicating test method data reproducibility, was the highest for TEE (>0.90) and EC (>0.90), followed by ED (0.61–0.66), and the lowest for APWA (0.41–0.45). In comparison to iPATD_CO_ measurements, TEE_CO_ (Figure 3A) and EC_CO_ (Figure 3B) consistently displayed underestimation (positive relative bias; slope < 1 about Y = X on regression line) across time points and during sequential blood volume manipulation. Conversely, ED_CO_ (Figure 3C) and APWA_CO_ (Figure 3D) consistently demonstrated significant overestimation of iPATD_CO_ measurements (negative relative bias; slope > 1 about Y = X on regression line).

**Table 2:** Percentage error (%), mean ± standard deviation (SD) of the relative bias (%), limits of agreement (%), and Lin’s concordance correlation coefficient (ρ_c_) for the cardiac output (CO) values measured using the test methods transesophageal echocardiography (TEE_CO_), arterial pressure waveform analysis (APWA_CO_), esophageal Doppler (ED_CO_), and electrical cardiometry (EC_CO_) in comparison with the reference method intermittent pulmonary artery thermodilution.

CO values from test method	Timepoint	Percentage error (%)	Relative bias (%) Mean ± SD	Limits of agreement (%)	Lin’s concordance (ρ_c_)
TEE_CO_	Pre-PLR_M_	27.1	14.2 ± 18.0	−21.1 to 49.5	0.94
	PLR_M_	27.2	10.4 ± 15.7	−20.4 to 41.1	0.93
	Post-PLR_M_	26.5	11.7 ± 14.0	−15.7 to 39.0	0.94
APWA_CO_	Pre-PLR_M_	64.2	−24.3 ± 43.3	−109.2 to 60.6	0.45
	PLR_M_	57.8	−26.0 ± 40.3	−105.0 to 53.0	0.42
	Post-PLR_M_	55.6	−34.5 ± 41.2	−115.2 to 46.2	0.41
ED_CO_	Pre-PLR_M_	26.8	−31.0 ± 17.6	−65.5 to 3.4	0.66
	PLR_M_	20.8	−31.1 ± 13.2	−57.0 to −5.1	0.61
	Post-PLR_M_	23.7	−34.4 ± 17.9	−69.5 to 0.8	0.64
EC_CO_	Pre-PLR_M_	24.4	3.1 ± 12.9	−22.1 to 28.4	0.93
	PLR_M_	23.1	3.8 ± 10.6	−16.9 to 24.5	0.92
	Post-PLR_M_	24.5	0.8 ± 14.4	−27.5 to 29.0	0.94

These data were obtained from six healthy, mechanically ventilated, isoflurane-anesthetized dogs undergoing sequential manipulation of blood volume: Step 1: baseline; Step 2: acute hemorrhage by withdrawing circulating blood volume (33 mL kg^−1^); Step 3: autologous blood transfusion; and Step 4: 20 mL kg^−1^ intravenous bolus of 6% hydroxyethyl starch (colloid). Data were collected at three timepoints in each step: immediately before performing the modified passive leg raise (PLR_M_) maneuver (Pre-PLR_M_), 5 min after PLR_M_ (PLR_M_), and 5 min after the abdomen and limbs were returned to the horizontal position (Post-PLR_M_).

**Figure 4:**
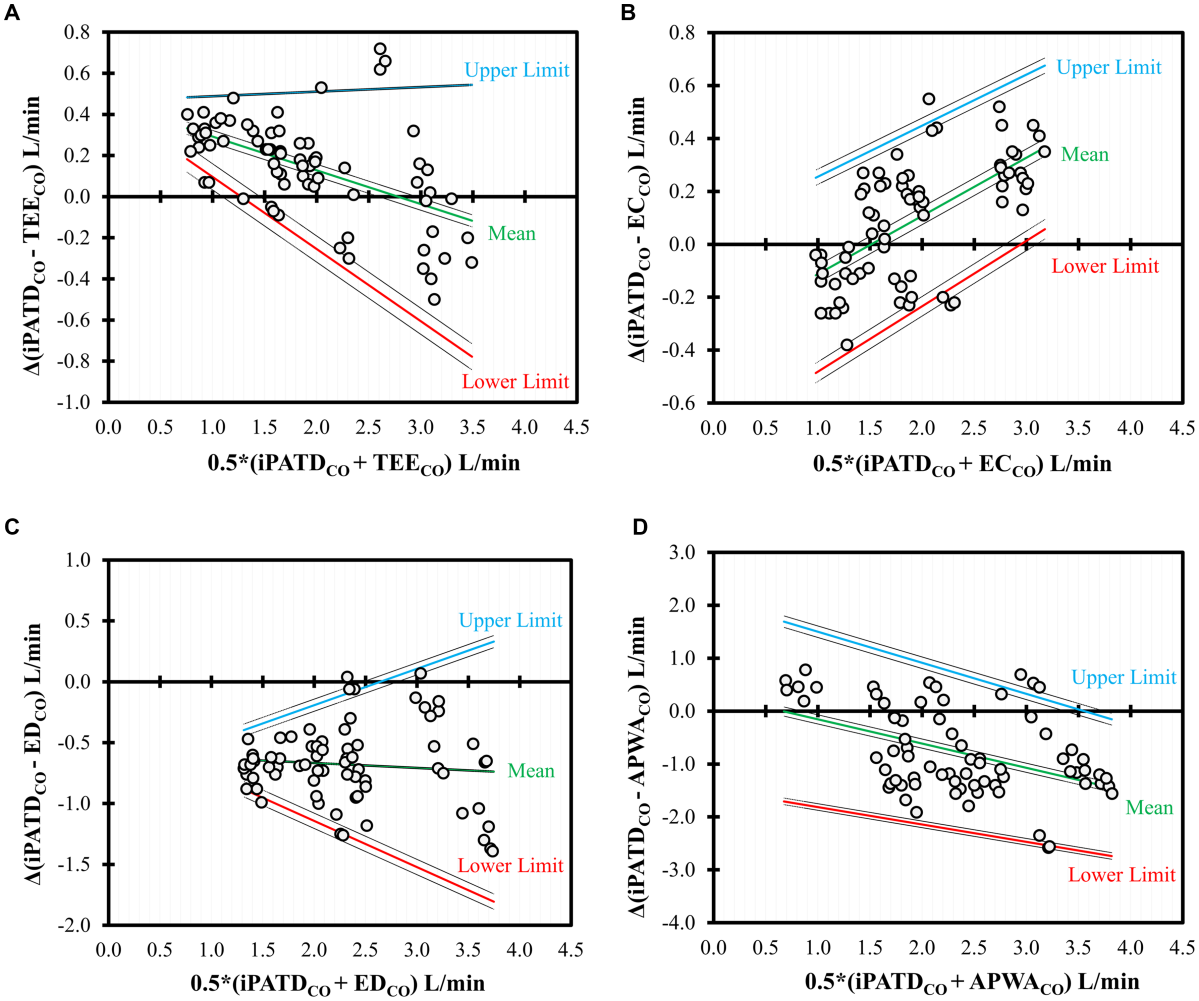
Bland–Altman analysis of the nonuniform differences in cardiac output (CO) measurements using **(A)** transesophageal echocardiography (TEE_CO_), **(B)** electrical cardiometry (EC_CO_), **(C)** esophageal Doppler (ED_CO_), and **(D)** arterial pressure waveform analysis (APWA_CO_) as compared with the reference method intermittent pulmonary artery thermodilution (iPATD_CO_) in six healthy, anesthetized dogs across three timepoints (before, during, and after the modified passive leg raise maneuver) and during sequential acute manipulations to the blood volume, which yielded 72 paired observations (circles) per test method. Each circle represents an individual comparison of the difference with the mean, and the central line represents the nonuniform mean bias of the difference. The solid lines indicate the mean (green) as well as the upper (blue) and lower (red) limits of agreement, and the dashed lines indicate the 95% confidence intervals around these values.

BA analysis for TEE_CO_ (Figure 4A) showed a negative trend (slope = −0.16; intercept = 0.45) between bias and average CO data, indicating underestimation for lower CO values and slight overestimation at higher CO values. EC_CO_ BA analysis (Figure 4B) revealed a positive trend (slope = 0.22; intercept = −0.33) between bias and average CO data, suggesting increased bias at higher CO values. Slight overestimation was observed for lower CO values, with slight underestimation at higher CO values. Overall, good agreement emerged between TEE, EC, and iPATD, with only a few observations falling outside the limits of agreement, and bias magnitude relatively small across a range of CO values. BA analysis for ED_CO_ (Figure 4C) showcased a very weak negative trend (slope = −0.04; intercept = −0.59) between bias and average CO data, suggesting uniform bias distribution across a broad CO range, with a relatively higher magnitude of consistent overestimation. APWA_CO_ BA analysis (Figure 4D) demonstrated a strong negative trend (slope = −0.46; intercept = 0.30) between bias and average CO data, indicating consistent overestimation across a wide CO range, with bias increasing at higher CO values. Consequently, only satisfactory agreement was observed between ED, APWA, and iPATD, with a few observations lying outside the limits of agreement.

**Figure 5:**
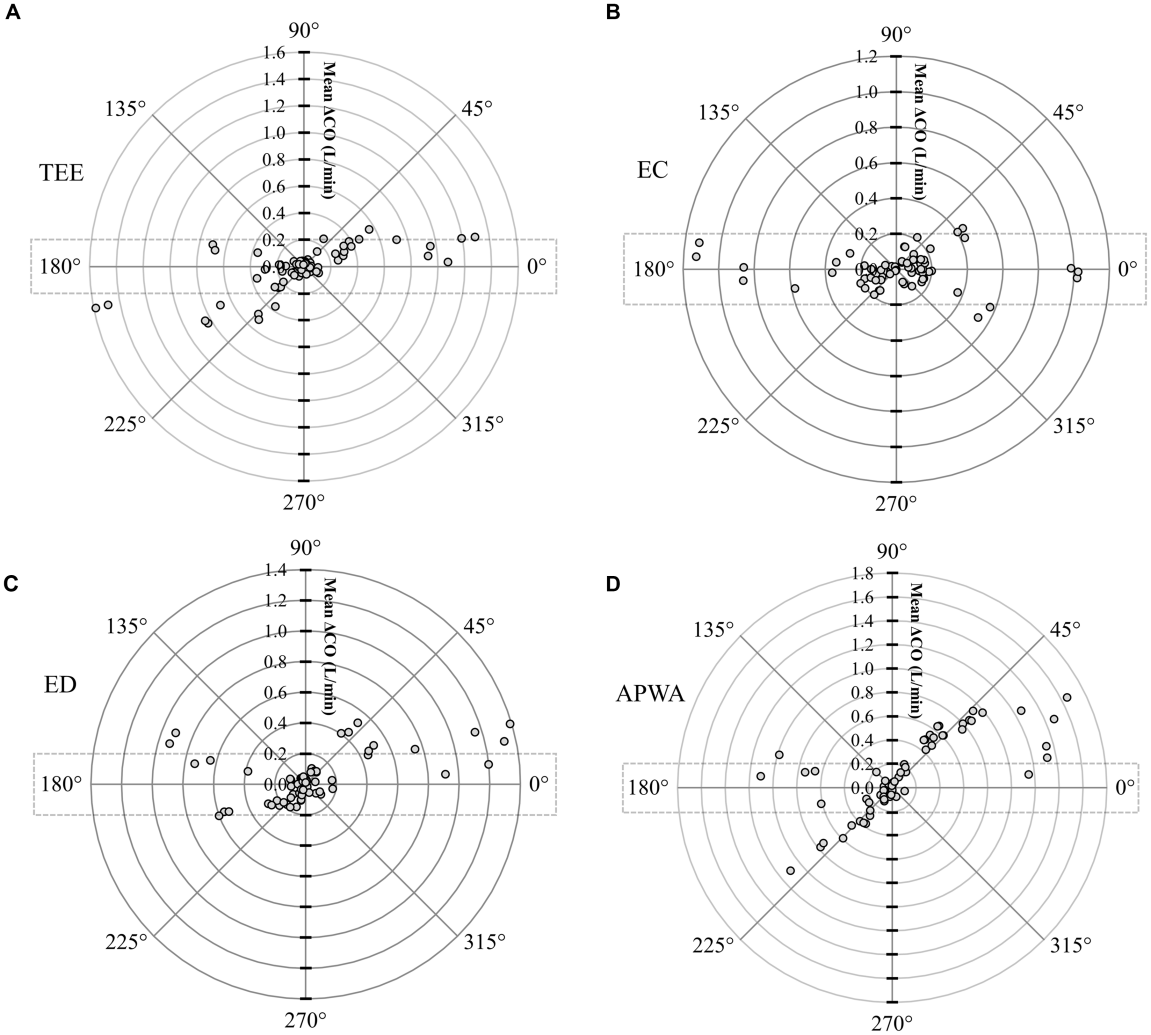
Polar plot representing changes in cardiac output (CO) measurements evaluated by **(A)** transesophageal echocardiography (TEE_CO_), **(B)** electrical cardiometry (EC_CO_), **(C)** esophageal Doppler (ED_CO_), and **(D)** arterial pressure waveform analysis (APWA_CO_) as compared with the reference method intermittent pulmonary artery thermodilution (iPATD_CO_) in six healthy, anesthetized dogs across three timepoints (before, during, and after the modified passive leg raise maneuver) and during sequential acute manipulations to the blood volume, thus yielding 72 paired observations (circles) per test method. Dotted lines represent 10% agreement boundaries (i.e., 10% = 0.198 Lmin-1 as mean iPATD_CO_ = 1.98 Lmin-1). The distance from the center represents the absolute values of the mean change in CO ([ΔiPATD_CO_ + ΔTest method CO]/2), and the angle from the horizontal 0° radial axis represents the disagreement. Polar plot analysis revealed a good trending pattern for TEE_CO_, EC_CO_, and ED_CO_ across the wide range of CO values, as <20% of the data points were located outside the limits of good agreement. By contrast, the trending ability of APWA_CO_ was poor, as >50% of the data points were located outside the limits of good agreement.

Polar plot analysis revealed strong trending patterns for TEE_CO_ (Figure 5A), EC_CO_ (Figure 5B), and ED_CO_ (Figure 5C), with <20% of data points situated outside the limits of good agreement (i.e., 10% = 0.198 Lmin-1 as mean iPATD_CO_ = 1.98 Lmin-1). Contrarily, the trending ability of APWA_CO_ was poor, with >50% of data points located outside the limits of good agreement, as depicted in the polar plot (Figure 5D).

## 4. Discussion

During the past two decades, the dependability of the passive leg-raising test has been substantiated across a diverse range of human patients, including those with sepsis in line with the Surviving Sepsis Campaign (45), and in patients with COVID-19 manifesting with acute respiratory failure (46). Furthermore, its predictive efficacy persists in patients featuring spontaneous ventilation, cardiac arrhythmias, and right ventricular failure (2, 47). A novel adaptation of the traditional passive leg raise has recently been applied to veterinary contexts, wherein both the pelvic limbs and caudal abdomen are elevated, prompting a significant redistribution of blood volume from the caudal region toward the central compartment. In healthy mechanically ventilated isoflurane-anesthetized pigs (21) and Beagle dogs (22), employing the PLR_M_ maneuver at angles of 15° and 30° accurately gaged FR during acute hemorrhagic shock, yielding an increase in iPATD_CO_ of more than 30% (pigs) and 28% (dogs), respectively. In instances of euvolemia and hypervolemia, marginal variations in CO were observed, indicating fluid nonresponsiveness. Remarkably, the percentage CO increase attributed to PLR_M_ surpassed the conventional 15% benchmark from human studies (2–4), highlighting the credibility of outcomes in these two animal studies (21, 22). In our investigation, as we examined the impact of PLR_M_ amidst moderate–severe hypovolemia, TEE, EC, and ED successfully diagnosed FR by detecting a > 15% CO elevation, akin to the reference iPATD method. Other hemodynamic variables derived from EC (such as SVV) and ED (such as SDV and PVV) exhibited close alignment with fluctuations in iPATD_CO_ values stemming from abrupt blood volume shifts and responses to the PLR_M_ maneuver. This correspondence harmonizes with previously reported canine literature (25, 27, 48–50). The TEE, ED, and EC displayed an acceptable percentage error range, while APWA exhibited the lowest Lin’s concordance. Generally, TEE and EC displayed good agreement with iPATD, maintaining a consistent trending pattern across a broad spectrum of CO values.

A comprehensive meta-analysis conducted on critically ill human patients, exploring the prognostic value of CO changes induced by the passive leg raising (at angles of 30–45°), reported pooled sensitivity and specificity values of 89.4 and 91.4%, respectively, based on the analysis of 351 fluid boluses. The pooled area under the curve stood at 0.95, with the prediction threshold for fluid responsiveness ranging from 8 to 15%. The alterations in CO resulting from the passive leg raise test correlated well with the CO increase facilitated by fluid infusion, effectively distinguishing between fluid responders and nonresponders (12). Correspondingly, Monnet et al. (14) identified 21 studies encompassing 991 adult patients, scrutinizing the hemodynamic effects of 995 fluid challenges. The study yielded a notably significant pooled correlation coefficient of 0.76 (*p* < 0.001) between CO readings influenced by the test and those by fluid administration. The optimal threshold mean was a CO elevation of more than 10 ± 2%, reflective of test performance. Across multiple clinical trials (12–15), primary variables for precise hemodynamic response detection encompassed CO, cardiac index, stroke volume, stroke volume index, or aortic blood flow. The techniques commonly employed for direct measurement of these variables involved calibrated pulse contour analysis, echocardiography, ED, iPATD, transpulmonary thermodilution, bioimpedance, bioreactance, and APWA. Therefore, the advanced hemodynamic monitoring techniques selected for our study were iPATD (considered the gold standard), TEE, EC, ED, and APWA, ensuring congruence with published human patient findings.

Minimally invasive TEE not only delivers precise, swift, and reliable CO measurements but also assesses derived parameters such as respiratory variations in superior and inferior vena cava diameter, maximal Doppler velocity in the LVOT, left ventricular ejection fraction, LVOT velocity–time integral, and ventricular end-diastolic area ratio. These parameters have demonstrated utility in investigating the effects of the passive leg raise test within human medicine (51). While TEE’s specific evaluation for FR in dogs has not been explored, its precision in CO estimation is well documented (52). Nonetheless, TEE does entail substantial training, skill, and high equipment costs, rendering it potentially less accessible at the bedside. Conversely, noninvasive EC is extensively employed in both adults and children owing to its simplicity, convenience, and continuous hemodynamic monitoring. However, its diagnostic value in the context of the passive leg raise test remains relatively unexplored. In pediatric patients undergoing cardiac surgery, EC-derived stroke volume and cardiac index were evaluated for FR prediction and effectiveness of the passive leg raise test, demonstrating accuracy in guiding circulatory function (53). Similarly, thoracic electrical bioimpedance cardiography exhibited good discriminatory ability in predicting responders to the passive leg raise in critical situations. Among key parameters, a secondary derivative of impedance wave slope displayed the highest predictive capacity for responder identification (54). It is essential to note that the EC technique’s Electrical Velocimetry™ model leverages the volume of electrically participating tissue based on anthropometric measures such as body mass and height, to estimate the electrically participating volume of the thorax. Predominantly influenced by patient weight, the mass-based volumetric equivalent of thoracic blood volume was determined using human subjects in both stable normal states and unstable cardiopulmonary disease states (55, 56). Consequently, the translation of this patient constant, derived from human data, to canines might account for potential errors reported in our EC analysis. Overall, the performance of TEE and EC in our study dogs indicated these methods could be used interchangeably with iPATD in assessing the PLR_M_ maneuver.

When interpreting ED_CO_ values, despite the percentage error remaining within acceptable bounds, this method consistently exhibited an overestimation of iPATD_CO_ measurements and displayed lower data reproducibility. Notably, the precision of measuring descending aortic blood velocity is significantly influenced by the alignment between the Doppler ultrasound beam and blood flow direction, termed the angle of insonation (57, 58). When employing ED, the sole approach to ensuring precise alignment lies in optimizing signal quality, manifested as a constant, distinct aortic waveform featuring the highest peak velocity, accompanied by a pronounced pitch “whip crack” sound. As the angle between the Doppler beam and blood flow widens, the inaccuracy in blood velocity measurements escalates due to the cosine error in the Doppler equation, coupled with deviation from the assumed angle of insonation (57–59). Furthermore, the ED veterinary monitoring system (CardioQ-EDMV+) lacks direct CO display, necessitating the use of the TEE-measured cross-sectional area of the LVOT to calculate ED_CO_, as previously described in dogs (39). In contrast to TEE, however, the ED_CO_ probe tip location, and consequently the flow traces’ origin, do not stem from the LVOT but from a segment of the descending aorta. Additionally, the potential discrepancy between the LVOT area and descending aorta area, the location where ED_CO_ traces were derived, could introduce variability and error into measurements. The ED human monitoring system (Cardio-Q ODM+) provides continuous CO measurements grounded in a human nomogram derived from patient age, height, and weight, correlating with the relationship between descending aortic blood velocity and iPATD_CO_ values (58). When used in animals, this human-based monitor might contribute to inaccurate CO evaluations.

Another CO measurement technique displaying substantial percentage error, low concordance, consistent iPATD_CO_ overestimation, and poor trending capacity was APWA. Several factors might have contributed to the heightened proportion of miscalculations seen with APWA. Lithium dilution serves as the preferred CO calibration method for the PulseCO algorithm, enabling beat-to-beat CO monitoring through a lithium-selective electrode sensor connected to a standard arterial line setup. Nondepolarizing muscle relaxants, however, can interact with the lithium sensor, leading to drift and calibration interference (60). Given that rocuronium bolus followed by constant rate infusion was administered throughout the study, its influence on APWA_CO_ values cannot be disregarded. During hemodynamic fluctuations, recalibration for the LiDCO system is advised with major shifts (35). In our study, recalibration was conducted only three times (before Steps 1, 3, and 4). We speculate that the PLR_M_ maneuver, particularly during hypovolemia, introduced significant hemodynamic shifts, evident in an iPATD_CO_ increase exceeding 30%. This could potentially contribute to APWA_CO_ errors. The PulseCO algorithm transforms the arterial pressure waveform into a volume-time waveform through autocorrelation and subsequently assesses the effective value using the root mean square approach. The “nominal stroke volume” is estimated during aortic ejection and equated with actual stroke volume through independent lithium dilution CO measurements (35, 61). An alternative scaling method entails employing a calibration factor based on a human nomogram derived from patient age, height, and weight. This accounts for vascular tree compliance specific to a given blood pressure (35, 62). However, direct application of this nomogram to veterinary species may not be appropriate. An inherent challenge of this method lies in the repeated lithium injections necessary for CO estimation, carrying a risk of lithium accumulation. It is worth noting that subpar performance of APWA has been reported in dogs (31, 63) and ponies (64) in previous studies.

Cardiac preload hinges upon the pressure gradient between mean systemic filling pressure and the right atrium, alongside vascular resistance. A considerable pressure gradient coupled with lower right atrial pressure corresponds to higher preload. Mean systemic filling pressure propels forward flow toward the right atrium, influenced by global blood volume and vascular compliance (9, 65). A specific resting fluid volume termed the “unstressed volume,” occupies the vascular bed without exerting force on vessel walls or contributing to venous flow. By contrast, the “stressed volume” stretches vessel walls, exerting an outward force that facilitates fluid movement from vessels (9, 65). The Frank-Starling cardiac function curve stipulates that when ventricular preload increases, the interaction between actin and myosin filaments intensifies, bolstering intrinsic cardiac contractility and CO. However, if myofilaments stretch beyond their maximum length, increased cardiac preload has no impact on CO, defining the ventricles as “preload unresponsive” (2–5). At this juncture, a fluid challenge might escalate stressed volume and mean systemic filling pressure, albeit to a lesser extent than right atrial pressure. This scenario risks volume overload (65). The passive leg-raising test rapidly and temporarily redistributes fluids within the vasculature, without altering total vascular volume. This maneuver taps into part of the resting volume in the venous reservoir of the lower body, converting unstressed volume into stressed volume (17). As stressed volume increases, mean systemic filling pressure surges, subsequently elevating right ventricular preload. If the right ventricle is fluid responsive, CO on the right side and left ventricular filling witness an increase (2, 16, 17). Conversely, in cases of fluid nonresponsiveness, the “self-transfusion” effect of leg raising boosts right atrial pressure but fails to elevate cardiac preload and CO. A significant advantage over fluid challenges is that the “virtual” bolus effects of leg raising can be swiftly reversed by returning the legs to a horizontal position (2, 17). The transient and reversible nature of the PLR_M_ maneuver hemodynamic effects was also evident in our study. Following acute hemorrhage, when the legs and caudal abdomen were repositioned (Post-PLR_M_), iPATD_CO_ measurements and variables such as SVV, SDV, and PVV values reverted to those recorded prior to the maneuver (Pre-PLR_M_).

The intensity of the hemodynamic response triggered by the passive leg raise test hinges on the extent to which unstressed volume from the lower body’s venous reservoir is redirected toward the cardiac ventricles, manifesting as a significant rise in venous return and CO. In human patients, this test can be executed using two methods: (1) beginning from a 45° semirecumbent position, then lowering the head and upper torso to lie supine while simultaneously elevating the legs at a 45° angle; and (2) initiating from a supine (horizontal) position and elevating the legs to a 30° to 45° angle (15). Upon comparison, researchers found that the supine position recruited approximately 300 mL of unstressed blood volume, whereas the semirecumbent position recruited approximately 450–500 mL of unstressed volume (2, 15, 19). The semirecumbent-initiated leg raise led to significantly higher central venous pressure and CO values compared with the supine-initiated leg raise, indicating the starting effect of the position on the preload change. In the 45° semirecumbent position, this phenomenon can be attributed to the greater transfer of unstressed venous blood volume from both the legs and the splanchnic compartment due to simultaneous trunk lowering and leg raising (2, 19). The adaptation of the passive leg raise test to animals drew inspiration from these human findings, while also accounting for potential differences in limb conformation, size, and blood volume distribution between humans and quadrupeds. Although the exact volume autotransfused by the PLR_M_ maneuver in pigs (21) and dogs (22) is undisclosed, the substantial iPATD_CO_ increase of ≈33% observed in our study during hypovolemia corroborates: (1) significant recruitment of unstressed blood volume from the pelvic limb and caudal abdomen venous reservoirs; (2) substantial conversion of unstressed blood to stressed blood by PLR_M_; and (3) preload responsiveness exhibited by both ventricles. The angle of inclination used for the PLR_M_ maneuver in our study was consistent with the established standard in dogs (22). In accordance with extensive human literature, it is recommended to assess the response to the passive leg raise within 90 s of test initiation due to its short-term circulatory effects; extending leg elevation does not preserve these effects (2, 16, 17). Data acquisition times (immediately before and 5 min after PLR_M_, and 5 min after returning the pelvic limbs and abdomen to their original positions) were aligned with the canine study, which demonstrated key hemodynamic changes within this timeframe. Continuous measurement of cardiovascular variables such as stroke distance, SDV, PVV, SVV, and EC_CO_ was maintained throughout the 5-min period. Based on data evaluation, we believe that the PLR_M_ maneuver induces a stable hemodynamic state lasting at least 5 min, although it remains unclear whether, after this period, the heart gradually adjusts to the new volume status or the effects start to diminish, potentially complicating their capture.

In the context of fluid administration decision-making, a pivotal question arises: does preload responsiveness invariably correlate with fluid resuscitation, while nonresponsiveness mandates fluid avoidance? Changes in cardiac contractility and afterload not only shift the Frank-Starling curve but also impact cardiac performance for a given preload. As a result, the ventricle does not operate on a single Frank-Starling curve; rather, a family of curves exists, characterized by inotropy and afterload (2, 5). Clinically, during acute absolute hypovolemia, a positive CO response to a fluid challenge can be anticipated, with the patient’s ventricles operating on the steeper portion of the Frank-Starling curve. However, fluid responsiveness does not always signify hypovolemia, and such patients may not require volume expansion. This concept was demonstrated in earlier canine studies (66, 67), where CO increased post-volume resuscitation in euvolemic, nonhypotensive, anesthetized dogs. Fluids augment the stressed venous volume, and considering the higher vascular capacitance of the venous system relative to the arterial system, a positive response to fluids that augment CO might be a normal physiological reaction. While the body is equipped to counter hypovolemia through homeostatic mechanisms, managing volume overload poses greater challenges. The absolute necessity for volume infusion must be assessed alongside the risks of fluid administration. The juxtaposition of the Frank-Starling curve and the Marik-Philips curve underscores the need to evaluate the effect of increasing preload and CO on extravascular lung water in fluid responders and nonresponders (8, 9). As a patient’s capacity to respond to fluids diminishes, extravascular lung water increases significantly due to elevated cardiac filling pressures and transmitted hydrostatic pressures (4).

Several factors may influence the response to the PLR_M_ maneuver, as indicated by human studies investigating the passive leg raise test (2, 12–15). Pain, autonomic stimulation, and vasoactive medications could alter the maneuver’s response. Patients with elevated intra-abdominal pressure might not be suitable candidates for the test, as PLR_M_ could exacerbate intra-abdominal hypertension and yield false results by interrupting caudal vena cava flow. Additionally, splanchnic blood volume recruited by the test could decrease in the presence of intra-abdominal hypertension (5, 16, 17). While keeping the thorax in the horizontal plane during PLR_M_ might reduce the risk of gastro-esophageal reflux, this requires further investigation. Furthermore, due to positional differences between PLR_M_ in our study and passive leg raise test in humans, it’s worth exploring whether PLR_M_ minimally affects intracerebral pressure and is safe to perform during head trauma. Considering that handling stress and anxiety can trigger sympathetic stimulation, utilizing PLR_M_ might not be feasible in awake canine patients, necessitating an examination of anesthetic and sedative influences on the test. Patients with pelvic and hindlimb fractures and hindleg amputations should not undergo PLR_M_. The response to the maneuver could also be altered during vasodilation and vasoconstriction due to variations in unstressed blood volume relative to stressed volume, as well as the availability of blood in the venous reservoir (2, 17). Executing PLR_M_ in the operating theater or during imaging procedures might not be practical for diagnosing FR. This simple, valuable, and cost-effective assessment tool could prove diagnostically valuable in clinical scenarios involving canine patients that are spontaneously breathing or mechanically ventilated, devoid of intra-abdominal hypertension, and experiencing: (1) arrhythmias; (2) hemodynamic collapse due to trauma; (3) absolute hypovolemia; (4) sepsis; or (5) admission to intensive care units, enabling repeated FR assessment without the risk of inducing pulmonary edema or cor pulmonale in potential fluid nonresponders.

Our study has several limitations. The small sample size is a notable constraint. The stepwise approach for acute blood volume manipulation was not randomized; its fixed sequence was crucial for detecting FR by PLR_M_ and the responsiveness of the tested CO methods. If hypervolemia preceded hypovolemia, crossover effects would significantly affect hemodynamics and our study data. The minimally invasive or noninvasive CO measurement techniques (EC, APWA, TEE, and ED) were selected based on current literature on CO monitoring in canines. It is acknowledged that while these techniques are less invasive and easy to learn, they may be costly for clinical practices and not universally available. Hence, other CO monitoring methods such as transpulmonary thermodilution, bioreactance, transpulmonary ultrasound dilution, and partial carbon dioxide rebreathing should be evaluated during PLR_M_ for their potential applicability in clinical settings. While integrating EC, ED, and APWA into veterinary species, careful scrutiny is needed regarding the mathematical models’ application based on human data for animals. The study involved healthy canines weighing approximately 10 kg placed in dorsal recumbency. Further clinical exploration is imperative to demonstrate the application of these CO measurement techniques during PLR_M_ in critically ill canine patients, encompassing different sizes, breeds, limb conformations, relative hypovolemia, and septic shock.

## 5. Conclusion

This study represents one of the first exploratory efforts to assess minimally invasive or noninvasive CO measurement techniques potentially substituting the invasive gold standard iPATD during PLR_M_ response evaluation in anesthetized healthy dogs undergoing acute blood volume manipulation. TEE, EC, and ED effectively diagnosed FR in hypovolemic dogs during this maneuver by detecting CO increases of >15%. Percentage error for TEE, ED, and EC fell within acceptable ranges, whereas it exceeded 55% for APWA. Moreover, TEE, EC, and iPATD exhibited good agreement, suggesting potential clinical applicability. Also, TEE_CO_, EC_CO_, and ED_CO_ displayed a satisfactory trending pattern across the range of CO values in our study dogs. Lin’s concordance was the highest for TEE and EC, followed by ED, and lowest for APWA. Further studies are essential to validate the constancy of TEE, EC, and ED performance across diverse clinical canine populations when applied during this maneuver.

## Data availability statement

The original contributions presented in the study are included in the article/supplementary material, further inquiries can be directed to the corresponding author.

## Ethics statement

The animal study was approved by Virginia Tech University-Institutional Animal Care and Use Committee (protocol number 20-235). The study was conducted in accordance with the local legislation and institutional requirements.

## Author contributions

VP: conception and study design, funding acquisition, execution and supervision of the study, animal care and management, training for the selected cardiac output techniques, data collection, data interpretation, original manuscript draft preparation, and artwork. NH-G and GM: data collection, review, and editing during manuscript preparation. SS: statistical analysis, data interpretation, review and editing during manuscript preparation, and figures for data presentation. All authors contributed to the article and approved the submitted version.

## Footnotes

^1^ https://andypope.info/charts/polarplot3.html
